# The Added Value of MRI-Based Targeted Biopsy in Biopsy-Naïve Patients: A Propensity-Score Matched Comparison

**DOI:** 10.3390/jcm13051355

**Published:** 2024-02-27

**Authors:** Gernot Ortner, Charalampos Mavridis, Veronika Fritz, Jörg Schachtner, Charalampos Mamoulakis, Udo Nagele, Theodoros Tokas

**Affiliations:** 1 Department of Urology and Andrology, General Hospital Hall i.T., 6060 Hall in Tirol, Austria; gerni_o@hotmail.com (G.O.); veronika.fritz@tirol-kliniken.at (V.F.); joerg.schachtner@tirol-kliniken.at (J.S.); prof@udonagele.at (U.N.); 2 Training and Research in Urological Surgery and Technology (T.R.U.S.T.)-Group, 6060 Hall in Tirol, Austria; ttokas@yahoo.com; 3 Department of Urology, University General Hospital of Heraklion, 71110 Heraklion, Greece; mamoulak@uoc.gr; 4 School of Medicine, University of Crete, 71003 Heraklion, Greece

**Keywords:** prostate biopsy, targeted biopsy, biopsy-naïve patients, MRI fusion biopsy, systematic biopsy, combined biopsy, clinically significant cancer, detection, added value, mpMRI

## Abstract

Background: Multiparametric Magnetic Resonance Imaging (mpMRI)-based targeted biopsy has shown to be beneficial in detecting Clinically Significant Prostate Cancer (csPCa) and avoiding diagnosis of Non-csPCa (ncsPCa); however, its role in the treatment of biopsy-naïve patients is still under discussion. Methods: After identifying predictors for the diagnosis of csPCa via Multivariate Logistic Regression Analysis (MLRA), a propensity-score (1:1 nearest neighbor) matched comparison was performed between a Systematic-Only Biopsy (SOB) cohort and a mpMRI-based Combined (systematic + targeted) Biopsy (CB) cohort from two tertiary urologic centers (SOB: Department of Urology, University General Hospital of Heraklion, University of Crete, School of Medicine, Heraklion, Crete, Greece; CB: LKH Hall in Tirol, Austria). Only biopsy-naïve patients were included in the study. The study period for the included patients was from February 2018 to July 2023 for the SOB group and from July 2017 to June 2023 for the CB group. The primary outcome was the diagnosis of csPCa (≥ISUP 2); secondary outcomes were overall cancer detection, the added value of targeted biopsy in csPCa detection, and the reduction in ncsPCa diagnosis with CB compared to SOB. To estimate the Average Treatment effect of the Treated groups (ATT), cluster-robust standard errors were used to perform g-computation in the matched sample. *p*-values < 0.05 with a two-sided 95% confidence interval were considered statistically significant. Results: Matching achieved well-balanced groups (each *n* = 140 for CB and SOB). In the CB group, 65/140 (46.4%) patients were diagnosed with csPCa compared to 44/140 (31.4%) in the SOB group (RR 1.48, 95%-CI: 1.09–2.0, *p* = 0.01). In the CB group, 4.3% (6/140) and 1.4% (2/140) of csPCa cases were detected with targeted-only and systematic-only biopsy cores, respectively. In the CB group, 22/140 (15.7%) patients were diagnosed with ncsPCa compared to 33/140 (23.6%) in the SOB group (RR = 0.67, 95% CI: 0.41–1.08, *p* = 0.1). When comparing SOB to CB (ATT), the marginal OR was 0.56 (95% CI: 0.38–0.82, *p* = 0.003) for the diagnosis of csPCa and 0.75 (95% CI: 0.47–1.05, *p* = 0.085) for the diagnosis of overall cancer (≥ISUP 1). Conclusion: The CB approach was superior to the SOB approach in detecting csPCa, while no additional detection of ncsPCa was seen. Our results support the application of mpMRI for biopsy-naïve patients with suspicions of prostate cancer.

## 1. Introduction

Prostate cancer is the most common cancer in men in Europe, accounting for 19.4% of the 45–64 age group and 25.3% in the over-65 age group [1]. In the United States, as of 2014, overall incidence is increasing by 3% per year, while there is an increase of 4.5% per year in diagnoses of advanced disease [2]. The widespread use of Prostate-Specific Antigen (PSA) testing, still a standard tool for diagnosing the disease, can explain these trends. Nevertheless, the sensitivity of PSA tests with values in the “grey zone” from 4 to 10 ng/mL reaches 93.1% with a specificity of 29.3% [3]. A major challenge that arises from typical screening is the overdiagnosis and overtreatment of Non-Clinically Significant Prostatic Cancers (ncsPCa), i.e., those with a Gleason score of six or an International Society of Urological Pathology (ISUP) grading equal to one (ISUP 1). The newer tools in our arsenal, such as the 4K, PHI, and Stockholm3 (STHLM3) tests, help to distinguish Clinically Significant PCa (csPCa) from ncsPCa [4,5]. However, they do not map the potentially clinically significant lesions. The most common method of confirming PCa is the Transrectal Ultrasound-guided Systematic Biopsy (TRUSB), which has many false-negative results in the grey zone [6]. Unfortunately, the prostate remains the only solid organ where biopsies are not targeted to a specific lesion [7]. A second problem that arises is the failure to detect csPCa. From 1990 onwards, Multiparametric Magnetic Resonance Imaging (mpMRI) appeared with the first published studies, and after a decade, the first MRI-guided prostate biopsy papers were published [8,9,10]. The Prostate Imaging–Reporting and Data System (PI-RADS) classification is used to distinguish lesions with a likelihood of csPCa in mpMRI. Initially, it was applied in cases of patients with a previous negative prostate biopsy performed by the conventional TRUSB method.

This data changed with the prospective paired-cohort PROMIS study, where the benefit of mpMRI before biopsy showed that the classical TRUSB method was inaccurate in detecting csPCa in biopsy-naïve patients [11]. In particular, the use of mpMRI findings in transrectal biopsies appeared to increase to 18% for the detection of csPCa. Furthermore, a significant proportion of the population (27%) could avoid unnecessary biopsies [11]. The results of the subsequent but randomized PRECISION study were similar [12]. The head-to-head comparison of targeted-only biopsies using mpMRI showed that it was superior to systematic biopsy only in detecting csPCa (adjusted absolute difference 12%, 95%-CI: 4–20, *p* = 0.005) [12]. It was also superior in not seeing ncsPCa (absolute difference 13%, 95%-CI: 7–19, *p* < 0.001) in biopsy-naïve patients [12]. Regardless of these landmark studies, the current landscape needs to be clarified. Recent data from the prospective MRI-FIRST study showed that performing targeted biopsies alone could misdiagnose 5.2% of patients with csPCa [13]. This high-level study showed the added value of combining systematic and targeted biopsies.

Before the reported pivotal studies of mpMRI in biopsy-naïve patients, two relevant comparative Randomized Controlled Trials (RCTs) had previously been published. The first showed that the detection of csPCa or ncsPCa did not differ between groups undergoing mpMRI–TRUSB cognitive fusion biopsies or randomized biopsies without MRI [14]. The second one had a similar design, although the fusion of the mpMRI and ultrasound images was software-based. In this study, the overall detection of PCa and the detection of csPCa was higher in the group that underwent mpMRI [15]. Although these studies were randomized, they were single-center, with a total number of patients of less than 250. The most recent and larger-scale randomized study among five centers showed that targeted biopsy with mpMRI was not inferior in lesions with PI-RADS of three or greater compared to systematically detecting csPCa. However, reduced detection of ncsPCa was identified in the mpMRI group (from 22% to 10%) [16].

Despite a strong recommendation for mpMRI evaluation in biopsy-naïve patients by the European Association of Urology, substantial heterogeneity exists nationally. Therefore, to reveal a potential real-world benefit in naïve patients, we aimed to compare the csPCa and ncsPCa detection rates between a mpMRI-based and a systematic-only biopsy approach between two tertiary urology centers.

## 2. Materials and Methods

### 2.1. Study Population

All biopsy-naïve men with a clinical suspicion of prostate cancer (for SB: PSA, DRE, for CB: PSA, DRE and mpMRI with at least one ≥ PI-RADS 3 lesion) and consecutive biopsies were included in this study. No specific exclusion criteria were defined.

Patients who underwent Systematic-Only Biopsy (Department of Urology, University General Hospital of Heraklion, University of Crete, School of Medicine, Heraklion, Crete, Greece—further referred to as “systematic only” biopsy (SOB) group) and patients who underwent an MRI-based lesion-targeted approach combined with systematic biopsy (LKH Hall in Tirol, Austria—further referred to as “combined biopsy” (CB) group) were included in the analysis. The study period for included patients was from February 2018 to July 2023 for the SOB group and from July 2017 to June 2023 for the CB group. No further stratifications for selecting specific subpopulations were applied.

### 2.2. Procedures

All patients underwent transrectal prostate biopsies under local anesthesia. Antibiotic prophylaxes were prescribed before intervention according to the institutions’ standards. For the CB group, the mpMRI imaging protocol (1.5–3 T machines) consisted of T2-weighted imaging obtained in at least two orthogonal planes, three-dimensional T2-weighted imaging, axial diffusion-weighted imaging obtained with multiple b-values, and axial contrast-enhanced dynamic imaging obtained after the injection of gadolinium contrast agent. All images were examined by two specialized uroradiologists with prior experience in prostate mpMRI imaging (>200 cases before the study started). The csPCa likelihood was assessed using the PI-RADS version 2.0 or 2.1 protocol for all patients. MpMRIs were obtained during a maximum period of eight weeks before CB. All externally obtained mpMRIs (minority of cases) underwent a secondary review by a specialized uroradiologist. Biopsies were only performed by urologists in both centers. In the CB group, two urologists (TT, VF) mainly performed the biopsies with an initial experience of >50 cases each before the study started. CMav and another urologist performed most of the biopsies for the SOB group with an initial experience of >150 cases each. Residents performed the rest under the supervision of the two urologists mentioned above. Targeted cores were obtained before systematic cores using a software-based approach (Biopsee™, MedCom GmbH, Darmstadt, Germany). Three to four cores were obtained for each lesion with a PI-RADS score ≥ 3, followed by 10–12 systematic cores. For the SOB group, between 7 and 35 cores were obtained. Cores were referred to histological analysis separately in the appropriate institution.

### 2.3. Data Retrieval and Processing

Prospectively held biopsy databases in both institutions were used for analysis. Only biopsy-naïve patients were selected for inclusion. Significant variables such as age, PSA, PSA-density (PSA-d), positive DRE, prostate volume, random and total cores, cancer yield, and ISUP grade were available for both institutions. For the CB group, further variables such as PI-RADS score, lesion diameter, lesion volume, targeted cores, cancer yield, ISUP grade, and minimal and maximal biopsy extent (% and mm of cancer present on core) according to the systematic and targeted cores were further available. The two principal investigators (GO and CMav) were responsible for the integrity of the data. Local ethics committees approved the study in both institutions (Department of Urology, University General Hospital of Heraklion, University of Crete, School of Medicine, Heraklion, Crete, Greece: decision number, including anonymous data sharing with LKH Hall in Tirol, Austria: 2882/2023; LKH Hall in Tirol, Austria: study number 1262/2022).

### 2.4. Outcomes

The primary outcome was the diagnosis of csPCa defined by ISUP ≥ 2 in any core. Secondary outcomes were the reduction in ncsPCa diagnosis, overall cancer (oaCa) diagnosis (≥ISUP 1), the diagnosis of cancer in random cores only, and the added value of targeted cores in the diagnosis of csCa and oaCa.

### 2.5. Statistical Analyses and Propensity Score-Matching

We reported results using descriptive statistics for continuous variables with mean + standard deviation (SD) and for dichotomous and categorical data with n/N (%). Continuous data were analyzed using a *t*-test or the Wilcoxon rank sum test, depending on the distribution homogeneity. Categorical data were analyzed with the Wilcoxon rank sum test and dichotomous data using either the Chi-square or Fisher’s exact test. *p*-values < 0.05 with a two-sided 95% confidence interval were considered statistically significant. Furthermore, Univariate Logistic Regression Analysis (ULRA) and Multivariate Logistic Regression Analysis (MLRA) (backward stepwise de-selection) were performed to calculate the predictors of the outcomes. Due to the heterogeneity between the groups, we used propensity score matching to estimate the effect (average treatment effect of the treated-ATT) of the biopsy approach (SOB vs. CB) on the diagnosis of csCa, accounting for confounding by the included covariates. A 1:1 nearest-neighbor propensity score-matching without replacement, with a propensity score estimated using logistic regression of the treatment (SOB vs. CB) on the covariates, was performed. We applied a 0.05 caliper to obtain well-balanced groups (Supplementary Figure S1). One hundred and forty patients for each SOB and CB group were matched. After matching, all standardized mean differences and variance ratios for the covariates were below 0.1 and between 0.89 and 1.02, respectively, indicating adequate balance (Supplementary Figure S2). To estimate the effect of the biopsy approach on the detection of csPCa, we fitted a binomial logistic regression model with csPCa as the outcome, the biopsy approach (SOB vs. CB) as the treatment selector, and covariates and their interaction as predictors and included the matching weights in the estimation. Using the avg_comparisons function in the marginal effects package, Cluster-robust standard errors were used to perform g-computation in the matched sample to estimate the ATT. Receiver Operating Characteristic (ROC) analysis was performed to test the performance of the fitted regression model, and the corresponding Area Under the Curve (AUC) was calculated. R version 4.0.3 (R Foundation for Statistical Computing, Vienna, Austria) was used for statistical analysis.

## 3. Results

### 3.1. Baseline Parameters

Data were available for 688 patients in the CB group and 196 in the SOB group. Table 1 presents an overview of baseline parameters for both groups, with significant differences in the major variables for predicting prostate cancer.

Further baseline parameters for the CB group regarding MRI-based information and targeted biopsy yield can be found in Supplementary Table S1. MRI revealed 9.9%, 66%, and 24% PI-RADS scores for three, four, and five lesions. Lesion locations were mainly in the peripheral (82%), followed by the transitional zone (18%) and anterior fibromuscular stroma (0.1%). The mean lesion diameter was 12.5 ± 7.3 mm, and the mean lesion volume was 0.64 ± 2.38 mL.

### 3.2. Detection of csCa and Overall Cancer before Propensity Score-Matching

Clinically significant cancer was identified in 39% of the CB group and 38% of the SOB group (*p* = 0.83). Overall, significantly more patients in the CB group were diagnosed with cancer compared to the SOB group (68% vs. 57%, *p* = 0.003). This difference was mainly based on additional ISUP 1 cancer diagnosed only with the targeted biopsy (an extra 9.4% in the CB group—Supplementary Table S1).

### 3.3. Identification of Predictors for csCa

For both the CB- and SOB-group, ULRA (Supplementary Tables S2 and S3) and MLRA (Supplementary Tables S4 and S5) were performed to reveal the predictors of csCa. For the CB group, MLRA revealed several predictors: age (OR 1.06, 95% CI: 1.03–1.10, *p* < 0.001), PSA-d ≥ 0.15 ng/mL^2^ (OR 4.69, 95% CI: 2.73–4.98, *p* < 0.001), and a PI-RADS score of five (OR 4.59, 95% CI: 1.63–13.8, *p* = 0.005, reference PI-RADS score three). Positive DRE did not show significance, yet it improved the overall predictability of the model (OR 2.17, 95% CI: 1.0–4.98, *p* = 0.057). For the SOB group, MLRA identified age (OR 1.06, 95% CI: 1.01–1.12, *p* = 0.02), PSA-d ≥ 0.15 ng/mL^2^ (OR 5.91, 95% CI: 2.64–13.7, *p* < 0.001), and positive DRE results (OR 7.51, 95% CI: 3.36–17.6, *p* < 0.001) as predictors.

### 3.4. Baseline Parameters of Matched Cohorts

After matching propensity scores using the predictors from the MLRA model for both groups as covariates (age, PSA-d, DRE-status), we obtained balanced groups with n = 140 patients for the SOB and CB groups (Supplementary Figures S1 and S2). The baseline characteristics of the matched cohorts are shown in Table 2. Further baseline parameters for the CB group regarding MRI-based information and targeted biopsy yield can be found in Supplementary Table S6. The MRI results revealed 7.1%, 64%, and 29% PI-RADS scores for three, four, and five lesions, respectively, and were comparable to the cohort before matching. Lesion locations were mainly in the peripheral (84%), followed by the transitional zone (16%). The mean lesion diameter was 12.5 ± 7.7 mm, and the mean lesion volume was 0.81 ± 3.09 mL.

### 3.5. Detection of csPCa, ncsPCa, and Overall Cancer after Matching

CB identified 15% more patients with csPCa than SOB (46% vs. 31%, RR 1.48, 95%-CI: 1.09–2.0, *p* = 0.01). In the CB group, the targeted biopsies only yielded an additional 4.3% of csPCa, whereas the systematic biopsies only yielded an extra 1.4% of csPCa. Out of 65 diagnosed csPCa cases in the CB group, 6/65 (9.2%), 2/65 (3.1%), and 57/65 (87.7%) were based on targeted-only, systematic-only, and targeted and systematic biopsy cores, respectively. In the CB group, 63/140 (45%) of csPCa would have been diagnosed if targeted biopsies had been performed alone compared to 59/140 (42.1%) with systematic biopsies alone (*p* = 0.72). In the CB group, 22/140 (15.7%) patients were diagnosed with ncsPCa compared to 33/140 (23.6%) in the SOB group (RR = 0.67, 95% CI: 0.41–1.08, *p* = 0.1). Of these 22 patients diagnosed with ncsPCa, 16/22 (72.7%) would have been diagnosed if a systematic biopsy was performed alone, and 13/22 (59.1%) would have been diagnosed if a targeted biopsy was performed alone (absolute risk difference: 13.6%).

### 3.6. Evaluation of Treatment Effect

ULRA (Supplementary Table S7) and MLRA (Table 3) were performed on the matched sample. The MLRA revealed several predictors for the diagnosis of csPCa: age (OR 1.07, 95% CI: 1.03–1.11, *p* < 0.001), PSA-d ≥ 0.15 ng/mL^2^ (OR 5.59, 95% CI: 2.67–10.3, *p* < 0.001) and positive DRE (OR 5.16, 95% CI: 2.67–10.3, *p* < 0.001) and the treatment (SOB OR 0.43, 95% CI: 0.23–0.78, *p* = 0.006, reference CB).

To account for the covariates’ influence, we estimated the ATT as described in the methodological section. Comparing SOB to CB, the marginal OR was 0.56 (95% CI: 0.38–0.82, *p* = 0.003) for the diagnosis of csPCa. However, we did not find a difference in the diagnosis rate of overall cancer (≥ISUP 1) when comparing SOB to CB with a marginal OR of 0.75 (95% CI: 0.47–1.05, *p* = 0.085).

## 4. Discussion

Since the beginning of the millennium, the approach of PCa diagnosis has slowly and steadily changed. Undoubtedly, mpMRI and, more recently, biparametric MRI (bpMRI) are the main contributors to progress as, for many decades, the prostate was the only solid organ that underwent random biopsies for cancer detection [7,8,17]. Naturally, the contribution of MRI techniques does not only concern the field of targeted biopsy but also the cost, which is estimated to be lower due to avoiding unnecessary biopsies, especially with the application of bpMRI [15,17].

Initially, our study showed the added value of MRI in biopsy-naïve patients in the matched cohort comparison; an additional 4.3% of csPCa with targeted-only cores were detected. Furthermore, the matched cohort comparison showed an absolute percentage difference of 15% in csPCa detection between the CB and SOB groups (46% and 31%, respectively, *p* = 0.01). This difference indicates that mpMRI contributes to the diagnostic accuracy of patients requiring prostate biopsy. All patients in the CB group had undergone mpMRI and had PI-RADS scores of ≥3. These findings align with studies showing the potential of mpMRI in detecting csPCa, including PRECISION and PROMIS [11,12,15]. Moreover, our results were slightly superior to the MRI-FIRST study, as we revealed csPCa in only 1.4% of samples with random biopsies. In the MRI-FIRST study, targeted biopsies missed 5.2% of patients with csPCa [13]. However, the Cochrane meta-analysis by Drost FH et al. showed a marginal benefit that was statistically insignificant for MRI usage in biopsy-naïve patients, with a pooled detection ratio of 1.05 (95% CI: 0.95 to 1.16; 20 studies) [18]. However, this meta-analysis did not include studies published after 2019.

The relevance of reducing insignificant cancer diagnoses in the CB group should also be mentioned here. As shown in Table 2, targeted-only biopsy detected 6/140 (4.3%) additional ISUP 1 cancers, compared to 9/140 (6.5%) and 7/140 (5.0%) with systematic only and systematic + targeted biopsy, respectively. This suggests the lowest absolute increase in nsPCa detection when performing targeted-only biopsies alone, with a small proportion (2/140 (1.4%)) of missed csPCa. Although the results of the present study regarding the diagnosis of ncsPCa are not impressive, they align with the existing literature. An RCT by Hugosson J. et al. showed that a targeted biopsy-only strategy reduced the risk of overdiagnosis by half [19]. At the same time, Klotz L. et al. identified a reduced detection of non-clinically significant cancers from 22% to 10% when performing targeted biopsies alone compared to a combined approach [16].

In the ULRA and MLRA models, PSA-d > 0.15 ng/mL^2^ was shown to be a strong predictor of csPCa presence in the matched cohort analysis with OR 6.2 and 5.59, respectively (95% CI 6.62–10.8 and 3.01–10.7). PSA-d is one of the strongest predictors of csPCa with a broad applicability [20]. In particular, it is commonly accepted that patients with PSA-d ≥ 0.15 ng/mL^2^ belong to the high-risk group for csPCa, whereas patients with PSA-d < 0.09 ng/mL^2^ are not likely to present with csPCa [21,22,23,24]. Furthermore, combining PSA-d with MRI findings may improve the negative predictive value of either Likert or PI-RADS scores [25,26]. It also appears to contribute to the detection of csPCa by enhancing MRI findings [27]. However, contradicting evidence showing that PSA-d does not improve the diagnostic performance of MRIs significantly and suggests rejecting the threshold of 0.15 ng/mL^2^ in cases where imaging findings are harmful to the presence of csPCa [28,29]. These conflicting results could be explained by the weakness of using PSA-d to indicate the presence of csPCa, in patients with a large-sized prostate or intraprostatic inflammation [22,30]. A study by Bruno SM et al. showed that slightly more than half of biopsy-naïve patients with PSA-d > 0.15 ng/mL^2^ did not present with csPCa, while most were without intraprostatic inflammation [30]. In the most recent European Association of Urology guidelines, the table from the meta-analysis by Schoots IG and Padhani AR linking the PI-RADS score to PSA-d has been suggested as a decision aid to decide whether a prostate biopsy should be performed. Based on this table, prostate biopsy is recommended even in patients with PI-RADS scores of 1–2 when PSA-d > 0.2 ng/mL^2^ and patients with a PI-RADS score 3 when PSA-d > 0.1 ng/mL^2^ [31].

Two other parameters that showed significant correlation with the presence of csPCa in the ULRA model were lesion diameter (cm) and lesion volume (mL), with OR 1.17 and 4.98, respectively (95% CI 1.1–1.26 and 2.07–14.3). A lesion diameter > 1 cm on MRI may predict the presence of csPCa, particularly in small prostates [24,32,33]. Also, it may be an independent risk factor for the extra-prostatic extension of the disease when the diameter exceeds 15 mm [34]. Regarding MRI lesion volume, studies have shown its correlation with PCa detection, specifically when it exceeds 1 mL [35,36]. Also, it has a higher diagnostic accuracy than PSA testing, even at smaller volumes [37].

Finally, it was noteworthy that overall PCa detection was higher in the CB group than in the SOB group (62% vs. 55%, respectively), although there was no statistically significant difference (*p* = 0.23). Variations in the incidence of PCa in Austria and Greece could explain this difference. In detail, the age-standardized rate of PCa per 100,000 people is 64.9 in Austria and 48.2 in Greece [38].

Our study has limitations by nature as it is retrospective. Despite not being randomized, we used propensity scores to match populations with good results. Also, the population compared after matching was relatively small, with 140 patients in each group out of 884 studied. Furthermore, in the CB group, only the overall targeted biopsy Gleason score was reported and linked to the lesion with the highest PI-RADS score in cases with two or more lesions on mpMRI. This adds a degree of bias to our results, as other lesions might have also yielded csPCa. Also, there was a selection bias in one group, as in the CB cohort, only patients with positive lesions were selected; patients with PI-RADS score ≤ 2 were excluded. This stratification potentially predisposed to a higher diagnosis of csPCa in this cohort; however, propensity score-matching should account for this. Furthermore, the consistency of mpMRI image quality was not assessed using the PI-QUAL scoring system as proposed by leading uroradiologists [39]. Additionally, both 1.5 T and 3 T MRI generators were used during the study, which could further impact csCa detection rates [40]. Finally, no comparison was made between the two cohorts based on the final histological report of patients undergoing radical prostatectomy. Thus, our data and analyses could change as Gleason upgrading is seen in a proportion of 33 to 49.3% of patients with ncsPCa after radical prostatectomy [41,42,43].

From a future perspective, we should also consider new tools to improve the detection rate of csPCa to a greater extent. Prata F et al. recently published promising results of a radiomic analysis with a clinically significant sensitivity of 91.5% and an area under the curve of 80.4% [44]. 

## 5. Conclusions

Undoubtedly, the development of MRI combined with widely used tests, such as PSA-d, has changed the landscape in the management of prostate biopsy candidates. Our retrospective study between two tertiary urologic centers used a propensity-score-matched comparison to demonstrate the added value of mpMRI-based targeted biopsy in biopsy-naïve patients. The targeted-only biopsy detected an additional 4.3% of patients with csPCa while showing the lowest absolute increase in ncsPCa detection compared to a systematic and compared biopsy approach. We have not yet reached the level of having clear and fundamental tactics of biopsy technique, nor the precise classification of those with ncsPCa. Therefore, large-scale RCTs are needed to compare the results of each method with the initial and post-radical prostatectomy biopsy.

## Figures and Tables

**Figure 1:**
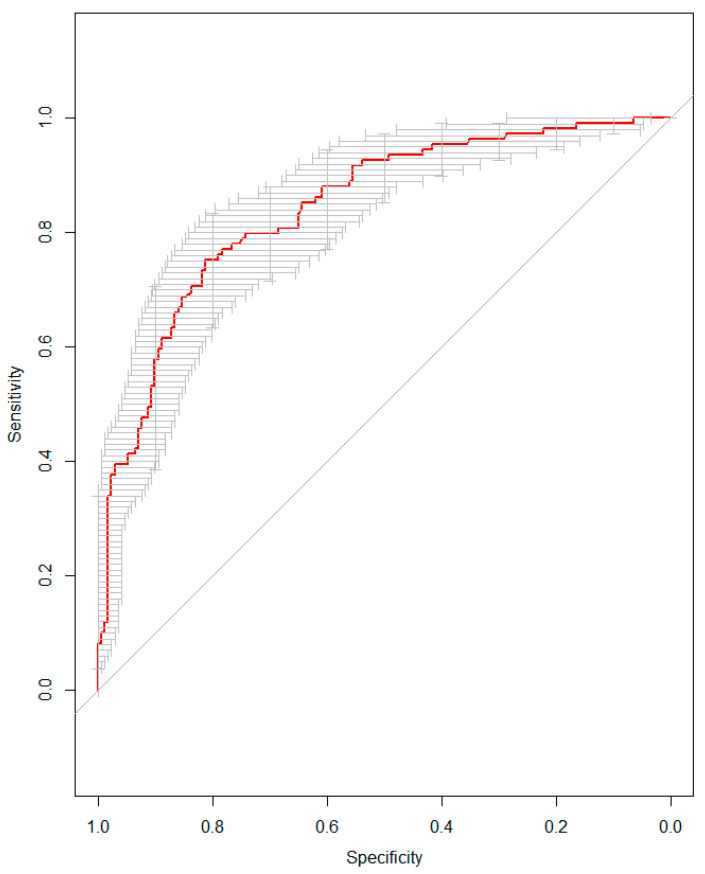
Receiver Operating Curve (ROC) analysis for the multivariate logistic regression model predicting Clinically Significant Cancer (csCa) after propensity score-matching. Treatment (SOB vs. CB) was adjusted for age, PSA density, and DRE result (all significant predictors, *p* < 0.005). An Area Under the Curve (AUC) of 0.84 (95% CI: 0.80–0.89) was obtained, indicating a good predictability of the model.

**Table 1:** Comparison of baseline characteristics of the Combined Biopsy (CB) and Standard-Only Biopsy (SOB) approach.

Baseline Parameters Unmatched Groups			
	CB, N = 688	SOB, N = 196	*p*-Value
Age	66.38 (8.96)	68.23 (8.49)	**0.008**
PSA [ng/mL]	6.99 (5.50)	13.14 (33.33)	**<0.001**
PSA-density [ng/mL^2^]	0.16 (0.13)	0.27 (0.67)	**0.003**
Positive DRE	104/688 (15%)	77/192 (39%)	**<0.001**
Prostate volume [mL]	50.41 (24.59)	52.34 (20.37)	**0.025**
Random cores	11.89 (1.03)	16.96 (4.29)	**<0.001**
Total cores	16.34 (1.52)	NA	
Target cores	3.73 (0.55)	NA	
% Positive random cores	17.82 (22.69)	25.80 (34.36)	0.18
% Positive target cores	41.25 (41.74)	NA	
csPCa overall	269/688 (39%)	75/196 (38%)	0.83
csPCa ONLY with target biopsies	42/688 (6.1%)	NA	
csPCa ONLY with random biopsies	23/688 (3.3%)	NA	
csPCa with random AND target	204/688 (30%)	NA	
Ca overall	471/688 (68%)	112/196 (57%)	**0.003**
Ca ONLY with target biopsies	65/688 (9.4%)	NA	
Ca ONLY with random biopsies	73/688 (11%)	NA	
Ca with random AND target	333/688 (48%)	NA	

Significant differences are highlighted in bold in the *p*-values column. Clinically Significant Prostate Cancer (csPCa) was defined by ISUP ≥ 2, and Cancer (Ca) was determined by ISUP ≥ 1—Digital Rectal Examination (DRE). Numbers are given in Mean (SD) for continuous and n/N (%) for binary data.

**Table 2:** Comparison of baseline characteristics of the Combined Biopsy (CB) and Standard-Only Biopsy (SOB) approach after propensity score matching.

Baseline Parameters Matched Groups			
	CB, N = 140	SOB, N = 140	*p*-Value
Age	69.13 (8.70)	68.33 (8.20)	0.43
PSA [ng/mL]	7.45 (5.56)	7.98 (5.43)	0.14
PSA-density [ng/mL^2^]	0.15 (0.11)	0.16 (0.12)	0.13
PSA-density-group			0.8
<0.15 ng/mL^2^	93/140 (66%)	95/140 (68%)	
>0.15 ng/mL^2^	47/140 (34%)	45/140 (32%)	
Positive DRE	40/140 (29%)	40/140 (29%)	>0.99
Prostate volume	57.18 (26.41)	53.01 (19.56)	0.28
Random cores	11.67 (1.53)	17.06 (4.43)	**<0.001**
Total cores	16.34 (1.77)	17.14 (4.40)	0.63
Target cores	3.63 (0.57)	NA	
% Positive random cores	18.99 (25.29)	20.33 (29.63)	0.8
% Positive target cores	40.57 (43.15)	NA	
csPCa overall	65/140 (46.4%)	44/140 (31.4%)	**0.01**
csPCa ONLY with target biopsies	6/140 (4.3%)	NA	
csPCa ONLY with random biopsies	2/140 (1.4%)	NA	
csPCa with random AND target	57/140 (40.7%)	NA	
Ca overall	87/140 (62.1%)	77/140 (55%)	0.23
Ca ONLY with target biopsies	12/140 (8.6%)	NA	
Ca ONLY with random biopsies	11/140 (7.9%)	NA	
Ca with random AND target	64/140 (45.7%)	NA	

Significant differences for PSA, PSA density, and DRE status are balanced. Significant differences are highlighted in bold in the *p*-values column. Clinically Significant Prostate Cancer (csPCa) was defined by ISUP ≥ 2, and Cancer (Ca) was determined by ISUP ≥ 1—Digital Rectal Examination (DRE). Numbers are given in Mean (SD) for continuous and n/N (%) for binary data.

**Table 3:** Results of the Multivariate Regression Analysis of the matched cohort.

Predictors of csPCa in the Matched Cohort			
	OR	95% CI	*p*-Value
Covariates			
CB	Reference	Reference	
SOB	0.43	0.23, 0.78	**0.006**
Age	1.07	1.03, 1.11	**<0.001**
PSA-density [ng/mL^2^]			
<0.15 ng/mL^2^	Reference	Reference	
>0.15 ng/mL^2^	5.59	3.01, 10.7	**<0.001**
Positive DRE	5.16	2.67, 10.3	**<0.001**

Odds Ratios (ORs) with 95% Confidence Intervals (CIs) are presented. Significant differences are highlighted in bold in the *p*-values column. Standard-Only Biopsy (SOB), Combined Biopsy (CB), and Digital Rectal Examination (DRE). The MLRA model achieved high predictability in the sensitivity analysis (AUC: 0.84, 95% CI: 0.80–0.89) (Figure 1).

## Data Availability

Data supporting the study’s results can be found in the Supplementary Materials.

## Supplementary Materials

The following supporting information can be downloaded at: https://www.mdpi.com/article/10.3390/jcm13051355/s1, Figure S1: Distribution of propensity scores and matching results using nearest neighbor matching with a caliper of 0.05. Figure S2: Covariate balance reflected by standardized mean difference and variance ratios before and after matching. Table S1: Further baseline parameters. Table S2: Univariate regression analyses for csPCa in the CB-group. Table S3: Univariate regression analyses for csPCa in the SOB group. Table S4: Multivariate regression analyses for csPCa in the CB-group. Table S5: Multivariate regression analyses for csPCa in the SOB group. Table S6: Further baseline parameters—propensity-score matched comparison. Table S7: Univariate regression analyses for csPCa in the matched cohort.
