# Supplementary material for: The Added Value of MRI-Based Targeted Biopsy in Biopsy-Naïve Patients: A Propensity-Score Matched Comparison
Source: J Clin Med. 2024 Feb 27;13(5):1355. doi: 10.3390/jcm13051355 (PMC10931596; doi:10.3390/jcm13051355)
Supplement: Supplementary file 1 [file jcm-13-01355-s001.zip › jcm-2846569-supplementary.pdf]

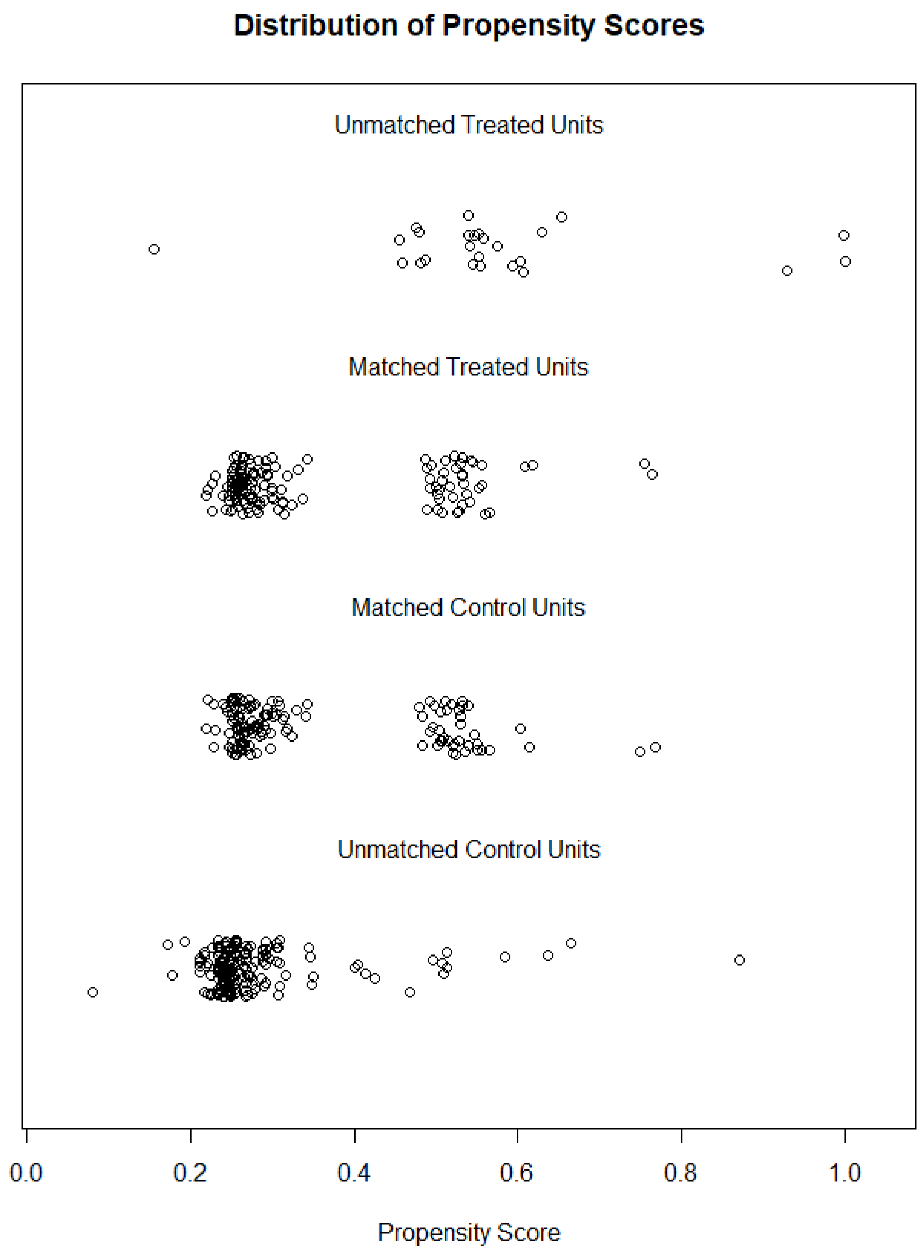


Supplementary Figure S1: Distribution of propensity scores and matching results using nearest neighbor matching with a caliper of 0.05.

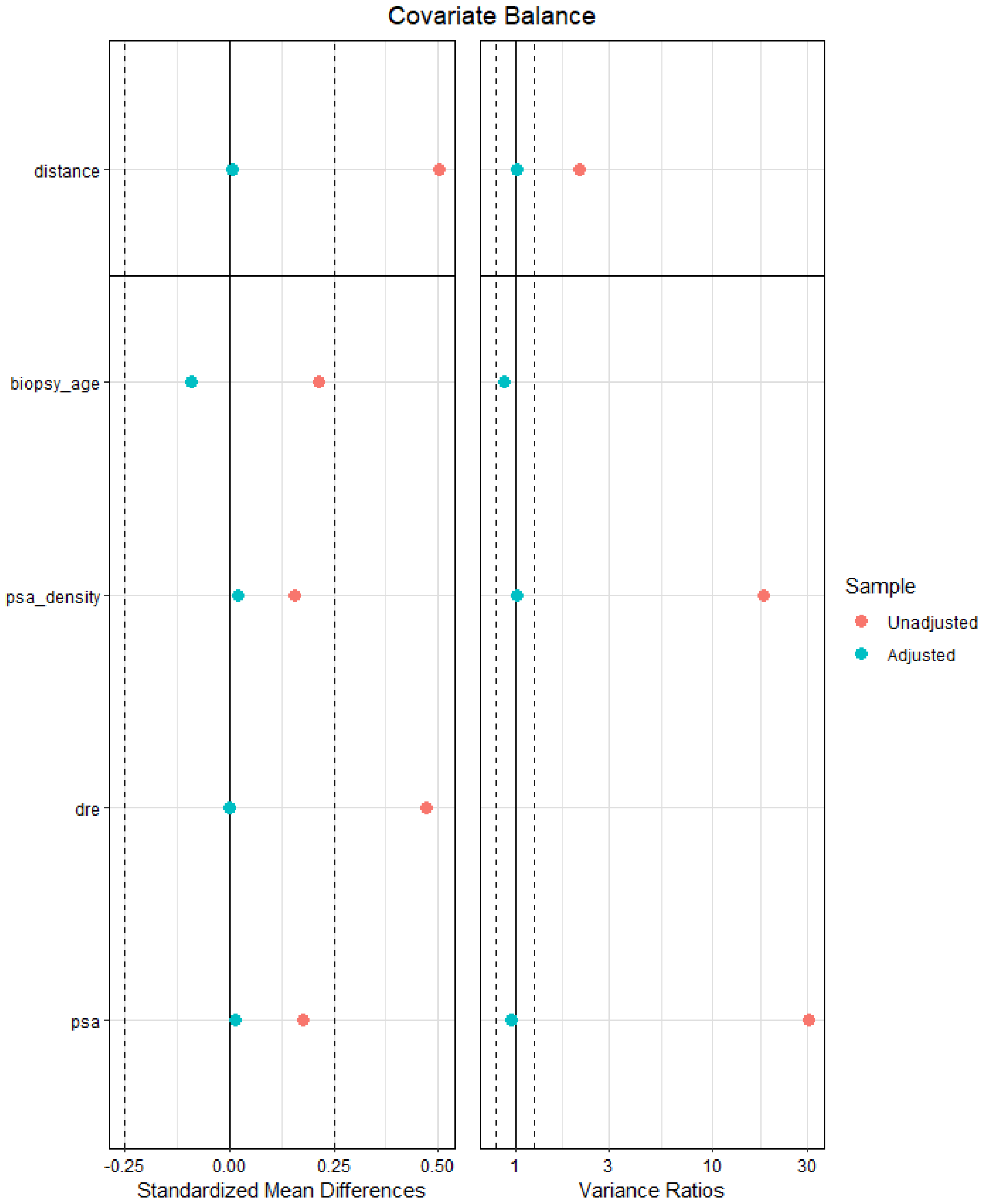


Supplementary Figure S2: Covariate balance reflected by standardized mean difference and variance ratios prior and after matching.

| Further baseline parameters | | | |
|---|---|---|---|
| | **CB, N = 688** | **SOB, N = 196** | **p-value** |
| **Lesion diameter [cm]** | 1.26 (0.73) | NA | |
| **Lesion volume [ml]** | 0.64 (2.38) | NA | |
| **Lesion PIRADS score** | | | |
| *3* | 68/688 (9.9%) | NA | |
| *4* | 453/688 (66%) | NA | |
| *5* | 167/688 (24%) | NA | |
| **Lesion location** | | | >0.99 |
| *AFS* | 1/688 (0.1%) | NA | |
| *PZ* | 564/688 (82%) | NA | |
| *TZ* | 123/688 (18%) | NA | |
| **Overall ISUP** | | | 0,35 |
| *5* | 8/688 (1.2%) | 9/196 (4.6%) | |
| *4* | 21/688 (3.1%) | 10/196 (5.1%) | |
| *3* | 52/688 (7.6%) | 18/196 (9.2%) | |
| *2* | 188/688 (27%) | 38/196 (19%) | |
| *1* | 202/688 (29%) | 37/196 (19%) | |
| *no cancer* | 217/688 (32%) | 84/196 (43%) | |
| **Random ISUP** | | | 0,32 |
| *5* | 7/688 (1.0%) | 9/196 (4.6%) | |
| *4* | 19/688 (2.8%) | 10/196 (5.1%) | |
| *3* | 46/688 (6.7%) | 18/196 (9.2%) | |
| *2* | 155/688 (23%) | 38/196 (19%) | |
| *1* | 179/688 (26%) | 37/196 (19%) | |
| *no cancer* | 282/688 (41%) | 84/196 (43%) | |
| **Target ISUP** | | | |
| *5* | 6/688 (0.9%) | NA | |
| *4* | 20/688 (2.9%) | NA | |
| *3* | 49/688 (7.1%) | NA | |
| *2* | 171/688 (25%) | NA | |
| *1* | 152/688 (22%) | NA | |
| *no cancer* | 290/688 (42%) | NA | |
| **Max. positive core target + random [%]** | 59.02 (29.43) | NA | |
| **Max. positive core target + random [mm]** | 6.86 (5.52) | NA | |
| **Max. core length target [%]** | 12.08 (4.58) | NA | |

Mean (SD); n/N (%)

Table S1: Further baseline parameters.

| Univariate regression analysis - csCa CB | | | |
|---|---|---|---|
| | **OR** | **95% CI** | **p-value** |
| **Age** | 1,07 | 1.05, 1.09 | **<0.001** |
| **PSA** | 1,13 | 1.09, 1.18 | **<0.001** |
| **PSA-density** | 1,676 | 268, 12,166 | **<0.001** |
| **PSA-density-group** | | | **<0.001** |
| <0.15 ng/ml² | — | — | |
| >0.15 ng/ml² | 3,76 | 2.70, 5.25 | |
| **Free PSA** | 1,01 | 1.00, 1.03 | 0,18 |
| **Prostate volume** | 0,99 | 0.98, 1.00 | **0,006** |
| **Seminal vesical invasion** | | | **0,001** |
| n | — | — | |
| y | 8,05 | 2.10, 52.6 | |
| **Rectal invasion** | | | 0,33 |
| n | — | — | |
| y | 3,13 | 0.30, 67.6 | |
| **Capsular invasion** | | | **0,012** |
| n | — | — | |
| y | 2,1 | 1.18, 3.78 | |
| **Total cores** | 0,95 | 0.85, 1.05 | 0,29 |
| **Target cores** | 1,05 | 0.89, 1.23 | 0,57 |
| **Random Cores** | 0,86 | 0.73, 1.00 | 0,054 |
| **Lesion diameter** | 1,07 | 1.04, 1.10 | **<0.001** |
| **Lesion volume** | 1,2 | 1.02, 1.59 | **0,019** |
| **DRE positive** | 4,86 | 2.47, 10.3 | **<0.001** |
| **PIRADS-score** | | | **<0.001** |
| 3 | — | — | |
| 4 | 2,43 | 1.31, 4.87 | |
| 5 | 7,32 | 3.76, 15.3 | |

OR = Odds Ratio, CI = Confidence Interval

Table S2: Univariate regression analyses for csPCa in the CB-group.

| Univariate regression analysis - csCa SOB | | | |
|---|---|---|---|
| | **OR** | **95% CI** | **p-value** |
| **Age** | 1,09 | 1.05, 1.14 | **<0.001** |
| **PSA** | 1,1 | 1.04, 1.17 | **<0.001** |
| **PSA-density-group** | | | **<0.001** |
| **<0.15 ng/ml** | — | — | |
| **>0.15 ng/ml** | 8,38 | 4.18, 17.5 | |
| **Free PSA** | 1,04 | 0.97, 1.12 | 0,27 |
| **Prostate volume** | 0,97 | 0.95, 0.99 | **<0.001** |
| **Random cores** | 0,97 | 0.91, 1.04 | 0,44 |
| **DRE positive** | 10,3 | 5.32, 20.9 | **<0.001** |

OR = Odds Ratio, CI = Confidence Interval

Table S3: Univariate regression analyses for csPCa in the SOB group.

| Multivariate regression analysis - csCa CB | | | |
|---|---|---|---|
| **Characteristic** | **OR** | **95% CI** | **p-value** |
| **Age** | 1,06 | 1.03, 1.10 | **<0.001** |
| **PSA-density-group** | | | |
| <0.15 ng/ml$^2$ | — | — | |
| >0.15 ng/ml$^2$ | 4,69 | 2.73, 8.22 | **<0.001** |
| **DRE positive** | 2,17 | 1.00, 4.98 | 0,057 |
| **PIRADS score** | | | |
| 3 | — | — | |
| 4 | 1,62 | 0.67, 4.25 | 0,3 |
| 5 | 4,59 | 1.63, 13.8 | **0,005** |

OR = Odds Ratio, CI = Confidence Interval

Table S4: Multivariate regression analyses for csPCa in the CB-group.

| Multivariate regression analysis - csCa SOB | | | |
|---|---|---|---|
| | **OR** | **95% CI** | **p-value** |
| **biopsy_age** | 1,06 | 1.01, 1.12 | **0,02** |
| **PSA-density** | | | |
| <0.15 ng/ml² | — | — | |
| >0.15 ng/ml² | 5,91 | 2.64, 13.7 | **<0.001** |
| **DRE positive** | 7,51 | 3.36, 17.6 | **<0.001** |

OR = Odds Ratio, CI = Confidence Interval

Table S5: Multivariate regression analyses for csPCa in the SOB group.

| Further baseline parameters - matched data | | | |
|---|---|---|---|
| | **CB, N = 140** | **SOB, N = 140** | **p-value** |
| **Lesion diameter [cm]** | 1.25 (0.77) | NA | |
| **Lesion volume [ml]** | 0.81 (3.09) | NA | |
| **Lesion PIRADS score** | | | |
| *3* | 10/140 (7.1%) | NA | |
| *4* | 89/140 (64%) | NA | |
| *5* | 41/140 (29%) | NA | |
| **Lesion location** | | | >0.99 |
| *PZ* | 117/140 (84%) | NA | |
| *TZ* | 22/140 (16%) | NA | |
| **Overall ISUP** | | | 0,094 |
| *5* | 1/140 (0.7%) | 4/140 (2.9%) | |
| *4* | 5/140 (3.6%) | 3/140 (2.1%) | |
| *3* | 15/140 (11%) | 13/140 (9.3%) | |
| *2* | 44/140 (31%) | 24/140 (17%) | |
| *1* | 22/140 (16%) | 33/140 (24%) | |
| *no cancer* | 53/140 (38%) | 63/140 (45%) | |
| **Random ISUP** | | | 0,67 |
| *5* | 1/140 (0.7%) | 4/140 (2.9%) | |
| *4* | 5/140 (3.6%) | 3/140 (2.1%) | |
| *3* | 12/140 (8.6%) | 13/140 (9.3%) | |
| *2* | 41/140 (29%) | 24/140 (17%) | |
| *1* | 16/140 (11%) | 33/140 (24%) | |
| *no cancer* | 65/140 (46%) | 63/140 (45%) | |
| **Target ISUP** | | | |
| *5* | 0/140 (0%) | NA | |
| *4* | 6/140 (4.3%) | NA | |
| *3* | 14/140 (10%) | NA | |
| *2* | 43/140 (31%) | NA | |
| *1* | 13/140 (9.3%) | NA | |
| *no cancer* | 64/140 (46%) | NA | |
| **Max. positive core target + random [%]** | 64.20 (31.86) | NA | |
| **Max. positive core target + random [mm]** | 7.69 (4.10) | NA | |

| | | | |
|---|---|---|---|
| **Max. core length target [%]** | 11.87 (4.60) | NA | |

Mean (SD); n/N (%)

Welch Two Sample t-test; Wilcoxon rank sum test; Pearson's Chi-squared test; Fisher's exact test; Kruskal-Wallis rank sum test

Table S6: Further baseline parameters – propensity-score matched comparison.

| Univariate regression analysis - csCa matched cohort | | | |
|---|---|---|---|
| | **OR** | **95% CI** | **p-value** |
| **Treatment** | | | **0,01** |
| CB | — | — | |
| SOB | 0,53 | 0.32, 0.86 | |
| **Age** | 1,09 | 1.06, 1.13 | **<0.001** |
| **Age-group** | | | **<0.001** |
| <60 | — | — | |
| 60-70 | 1,58 | 0.74, 3.60 | |
| 70-80 | 4,03 | 1.92, 9.02 | |
| >80 | 6,92 | 2.30, 22.7 | |
| **PSA** | 1,1 | 1.04, 1.16 | **<0.001** |
| **PSA-density-group** | | | **<0.001** |
| <0.15 ng/ml² | — | — | |
| >0.15 ng/ml² | 6,2 | 3.62, 10.8 | |
| **Free PSA** | 1 | 0.98, 1.02 | 0,87 |
| **Prostate volume** | 0,95 | 0.93, 0.97 | **<0.001** |
| **Total cores** | 0,98 | 0.91, 1.06 | 0,67 |
| **Target cores** | 0,98 | 0.73, 1.33 | 0,92 |
| **Random cores** | 0,95 | 0.89, 1.01 | 0,1 |
| **Lesion diameter** | 1,17 | 1.10, 1.26 | **<0.001** |
| **Lesion volume** | 4,98 | 2.07, 14.3 | **<0.001** |
| **Lesion location** | | | 0,28 |
| PZ | — | — | |
| TZ | 0,62 | 0.23, 1.57 | |
| **Positive DRE** | 7,7 | 4.35, 14.1 | **<0.001** |
| **PIRADS-score** | | | **<0.001** |
| 3 | — | — | |
| 4 | 2,03 | 0.47, 14.0 | |
| 5 | 16,5 | 3.39, 125 | |

OR = Odds Ratio, CI = Confidence Interval

Table S7: Univariate regression analyses for csPCa in the matched cohort.
